# Editorial: Post-harvest Diseases of Fruit and Vegetable: Methods and Mechanisms of Action

**DOI:** 10.3389/fmicb.2022.900060

**Published:** 2022-04-13

**Authors:** Khamis Youssef, Antonio Ippolito, Sergio Ruffo Roberto

**Affiliations:** ^1^Agricultural Research Center, Plant Pathology Research Institute, Giza, Egypt; ^2^Agricultural and Food Research Council, Academy of Scientific Research and Technology (ASRT), Cairo, Egypt; ^3^Department of Soil, Plant and Food Science, University of Bari Aldo Moro, Bari, Italy; ^4^Agricultural Research Center, State University of Londrina, Londrina, Brazil

**Keywords:** shelf-life, fruit, post-harvest pathology, vegetables, fungi

## Introduction

Post-harvest losses of fruit and vegetables are very high and sometimes reach 50% and more in some developing countries due to pathological and physiological deterioration (Smilanick et al., 2006). This loss is due to inappropriate handling and lack of right methods and means to prevent diseases. Fruits and vegetables are susceptible to severe losses caused by several plant pathogenic fungi including *Botrytis cinerea, Alternaria alternata, Penicillium italicum, P. digitatum, P. expansum, Monilia fructicola, M. laxa, Colletotrichum gloeosporioides, Rhizopus stolonifer, Botryiodiplodia theobromae etc.*, after harvest. Chemical fungicides are the primary means to control such diseases. However, several constrains have limited their use including fungicide resistance, market pressure regarding residues and concerns of environmental and human health (Hashim et al., 2019). In this context, safe alternative means to control post-harvest diseases of fruits and vegetables are needed to be developed (Lachhab et al., 2015; Salem et al., 2016). Several investigations have documented the strong antimicrobial activity of various alternatives including biological control using antagonistic microorganisms, physical means such as low temperatures, modified and controlled atmospheres, heat, irradiation, and generally regarded as safe (GRAS) substances such as salts, sanitizers, plant extracts, and essential oils. Also, many efforts have been done to understand their mode of action to improve their use, especially at large scale in the field.

The aim of this Research Topic was to present the latest results of controlling post-harvest diseases of fruit and vegetables using new alternative control means and understanding their mechanism of action. Eleven articles were accepted for this Research Topic dealing with table grapes, strawberries, avocados, mangoes, papayas, apples, and pears. In this context, Godana et al. investigated the mechanism by which *Pichia anomala* induced with chitosan (1% w/v) controls blue mold disease in table grapes caused by *Penicillium expansum*. The results of the study showed that chitosan (1% w/v) significantly increased the yeast β-1,3-glucanase, catalase (CAT), and malondialdehyde (MDA) activities. Furthermore, *P. anomala* alone or induced with chitosan significantly increased the table grapes enzymatic activities of Polyphenol oxidase (PPO), phenylalanine ammonia-lyase (PAL), peroxidase (POD), and catalase compared to the control. The RT-qPCR results also confirmed that the genes of these major disease defense enzymes were up-regulated when the table grapes were treated with *P. anomala*.

In China, as Novel Loop-Mediated Isothermal Amplification Detection Methods, Fu et al. isolated two bacterial strains (*Pseudomonas aeruginosa* and *Serratia marcescens*) from rotten post-harvest Fuzi. Two loop-mediated isothermal amplification (LAMP) methods targeting the gyrase B subunit (gyrB) gene of *P. aeruginosa* and the phosphatidylinositol glycan C (pigC) gene of *S. marcescens* were successfully developed, and it was found that the target genes were highly specific to the two pathogens.

Morales-Cedeño et al. evaluated the role of strain SER3 from the recently discovered *Rouxiella badensis* as a biocontrol agent. SER3 was isolated from the phyllosphere of decaying strawberry fruit (*Fragaria* × *ananassa* Duch.) and showed different grades of antagonism against 20 fungal pathogens of berries, based on confrontation assays, due to the action of its diffusible and volatile compounds. A comparison of the genomic properties of *R. badensis* SER3 and other close bacterial relatives showed several genes with potential functions in biocontrol activities, such as those encoding siderophores, non-ribosomal peptide synthetases, and polyketide synthases.

In a scientific collaboration between South Africa, Turkey and Italy Sivakumar et al. developed a comprehensive review on the impact of edible coatings, essential oils, and their nano formulations on post-harvest decay anthracnose of avocados, mangoes, and papayas. The authors summarized and analyzed the recent advances and trends in the use of these alternative post-harvest treatments on anthracnose decay in the mentioned fruit crops.

In apples, Deng et al. verified the use of sodium hydrosulfide against *Penicillium expansum* and it promoted the synthesis of endogenous hydrogen sulfide (H_2_S), hydrogen peroxide (H_2_O_2_), and nitrogen oxide (NO). Sodium hydrosulfide treatment enhanced the activities of phenylalanine ammonia-lyase, cinnamate 4-hydroxylase, p-coumarate:coenzyme A ligase isoenzymes, caffeoyl-CoA-O-methyltransferase, caffeic acid-O-methyltransferase, ferulic acid-5-hydroxylase, cinnamyl-CoA reductase, and cinnamyl-alcohol dehydrogenase. In Belgium, in the same fruit crop, Naets et al. verified that the early activation of the mevalonate pathway in fruits challenged by *Botrytis cinerea* correlated with reduced susceptibility during post-harvest storage. The authors demonstrated a clear transcriptional activation of secondary metabolism and a correlation between the early transcriptional activation of the mevalonate pathway and reduced susceptibility, expressed as a reduction in resulting lesion diameters. Also in apples, Sun, Liu, et al. used *Serratia plymuthica* to suppress apple ring rot on post-harvest apple fruit caused by *Botryosphaeria dothidea*. The treatment significantly reduced the titratable acidity content, enhanced the soluble sugar content, vitamin C, and their ratio, and maintained the firmness of the fruits. In another study on apples, Yuan et al. investigated the role of *Bacillus velezensis* Strain P2-1 as a biocontrol agent against apple post-harvest decay caused by *Botryosphaeria dothidea*. The authors summarized that PCR and qRT-PCR assays revealed that strain P2-1 harbored the gene clusters required for biosynthesis of antifungal lipopeptides and polyketides. Sun, Duan, et al. used dimethyl trisulfide to manage ring rot disease. The authors concluded that soluble sugar, vitamin C, and soluble sugar/titratable acidity ratio of the dimethyl trisulfide-treated fruit were significantly higher as compared to the control. Finally, in apple cv. Fuji, Ackah et al. summarized that chitosan accelerated apple wound healing by activating the phenylpropanoid pathway and stimulating enzymatic browning of wounds.

Some interesting results on “Doyenne du Comice” pear were obtained by Zhang et al. 1-methylcyclopropene was highly effective in reducing disease incidence and induced multiple changes of the fungal and bacterial microbiota. This study was the first comprehensive analysis of the microbiome response to 1-methylcyclopropene in post-harvest pear fruit, and reveals the relationship between fruit decay and microbial composition in pear fruit.

## Author Contributions

KY, SR, and AI: writing—review and editing. All authors contributed to the article and approved the submitted version.

## Conflict of Interest

The authors declare that the research was conducted in the absence of any commercial or financial relationships that could be construed as a potential conflict of interest.

## Publisher's Note

All claims expressed in this article are solely those of the authors and do not necessarily represent those of their affiliated organizations, or those of the publisher, the editors and the reviewers. Any product that may be evaluated in this article, or claim that may be made by its manufacturer, is not guaranteed or endorsed by the publisher.

## References

[B1] Hashim A. F. Youssef K. Abd-Elsalam K. A. (2019). Ecofriendly nanomaterials for controlling gray mold of table grapes and maintaining postharvest quality. Euro. J. Plant Pathol. 154, 377–388. 10.1007/s10658-018-01662-2

[B2] Lachhab N. Sanzani S. M. Fallanaj F. Youssef K. Nigro F. Boselli M.. (2015). Protein hydrolysates as resistance inducers for controlling green mould of citrus fruit. Acta Horticult. 1065, 1593–1598. 10.17660/ActaHortic.2015.1065.203

[B3] Salem E. A. Youssef K. Sanzani S. M. (2016). Evaluation of alternative means to control postharvest Rhizopus rot of peaches. Sci. Horticult. 198, 86–90. 10.1016/j.scienta.2015.11.013

[B4] Smilanick J. L. Brown G. E. Eckert J. W. (2006)“The biology control of postharvest diseases,” in Fresh Citrus Fruits, 2nd edn, eds Wardowski W. F. Miller W. M. Hall D. J. Grierson W. (Longboat Key, FL; Florida Science Source Inc.). p. 339–96.

